# Why Another Cancer Journal?

**DOI:** 10.3390/cancers1010001

**Published:** 2009-07-24

**Authors:** Robert H. Weiss

**Affiliations:** *Editor-in-Chief*, Division of Nephrology, Department of Internal Medicine, Genome and Biomedical Sciences Building, Room 6312, University of California, Davis, One Shields Ave., Davis, CA 95616, USA; E-mail: rhweiss@ucdavis.edu; Tel.: 530-752-4010; Fax: 530-752-3791

Why do we need yet another cancer journal? An excellent question, of course, in light of the profusion of general as well as specialized cancer journals in recent times. An easy answer would simply be to say that this journal will be better than the rest; however a less glib (and probably more accurate) response is simply that there is currently an explosion of new techniques and approaches in the field of cancer biology, and that the new, and sometimes unusual and unorthodox, data so generated demands an open-access and unrestricted forum for its dissemination. This was the impetus for starting the journal which you are now reading.

I was asked to be *Editor-in-Chief* of this new journal due perhaps to my efforts and publications in a relatively novel field which has compelling applications in cancer: metabolomics. While I have worked in “classical” cell signaling for many years, both in vascular and kidney as well as in cancer biology, I have more recently been attracted to the “omics” fields as applied to oncology, not just because it’s trendy and many other people are doing it, but because in this era of mass data generation and handling, it now appears (to me at least) that these fields in fact hold the keys to modern diagnosis as well as any future “personalized” therapy of cancer. And, because “omics” requires a team approach since nobody alone can be expert in all of its aspects (analytical chemistry, cell biology, bioinformatics, statistics, *etc.*), I am able to dabble in this area due to the willingness of my friends colleagues to help me as well as to educate (and put up with) my classically trained research team. It seems to me that the problem of cancer will be solved not just by the use of high powered computers available today, but also by that all-important team approach to cancer research: there is simply too much for any one individual to know about the complexity of such a complex disease.

We will be starting this new journal by publishing a special issue on Biomarkers later this year. This is a topic of prime importance to oncology which to date has been woefully under-investigated, although this appears to be changing at least in the US where the NIH is now convinced that the “fishing expedition” (which is a necessary component of “omics” research) is indeed a viable approach when done properly, and is thus promoting the field; this last fact is something to which I can attest by my good fortune in receiving three US federal grants for my metabolomics research.

It is undeniably clear that cancers can be best treated when discovered extremely early, even before any phenotypic or macroscopic changes have occurred, and at present (with a few weak exceptions) the disease is only treated when it shows up at the macro-level. For example, lung cancer is usually diagnosed by the patient spitting up blood or incidentally on chest x-ray, and diseases such as pancreatic, ovarian, and kidney cancer are generally diagnosed at horrifying late stages when it is frequently too late for effective therapy. The function of an ideal molecular biomarker is to utilize the genotypic and subsequent early molecular changes produced by a tumor (or by the milieu that will produce a tumor) to “catch” that cancer before it has a chance to gain a foothold in the body. This, of course, is a tall order and has really not been achieved satisfactorily by any marker as yet in any cancer; however, we will get there, most likely by exploiting “omics” technology.

It is also clear that there are many novel approaches to cancer therapy which include natural products. These compounds are expected to exist based on the simple fact that plants also have to protect themselves from excessive and/or unregulated growth, and those with the most successful mechanism for doing this have been more successful in evolution. Some of these plant-derived compounds have for centuries formed the basis of chemotherapeutics which are still in use, but others have yet to be discovered or proven. While many editorial staff in the “traditional” oncology publications look askance at these submissions, we plan to keep an open mind.

Many readers have no doubt been frustrated by the poor availability of an interesting article due to the necessity of having an expensive subscription to gain access to it. And there are those times when you are on the road and need access to an article but can’t get it, even if your institution has a license, because you are on your own computer! Cancers will be freely available on-line so that this will never be a problem. While certainly not the only such journal, Cancers is one of the few in the field of oncology. Given the extensive data both produced and required for hypothesis-testing in the new “omics” fields, the lack of length restriction in our journal will facilitate the publication of the most extensive studies.

In launching this new journal, we are hopeful that we can attract the best and most novel manuscripts which encompass many of the as-yet experimental oncology fields as well as the traditional areas of research in cancer biology and therapy. Those who submit to this journal can be sure that their accepted study will be disseminated quickly, broadly, and in its entirety to anyone anywhere who has internet access. We hope that you can join us in ensuring the success of this new journal.

